# Association between Obesity and Intake of Different Food Groups among Japanese with Type 2 Diabetes Mellitus—Japan Diabetes Clinical Data Management Study (JDDM68)

**DOI:** 10.3390/nu14153034

**Published:** 2022-07-24

**Authors:** Mariko Hatta, Chika Horikawa, Yasunaga Takeda, Izumi Ikeda, Sakiko Yoshizawa Morikawa, Noriko Kato, Mitsutoshi Kato, Hiroki Yokoyama, Yoshio Kurihara, Hiroshi Maegawa, Kazuya Fujihara, Hirohito Sone

**Affiliations:** 1 Department of Hematology, Endocrinology and Metabolism, Faculty of Medicine, Niigata University, Niigata 951-8510, Japan; marichocolate.coffee02@gmail.com (M.H.); horikawa@unii.ac.jp (C.H.); mr2.yac@gmail.com (Y.T.); ikdizm12@gmail.com (I.I.); sakiko.1211@gmail.com (S.Y.M.); sone@med.niigata-u.ac.jp (H.S.); 2 Saiseikai Niigata Hospital, Niigata 950-1104, Japan; 3 Department of Health and Nutrition, Faculty of Human Life Studies, University of Niigata Prefecture, Niigata 950-8680, Japan; 4 Department of Food Science and Dietetics, Faculty of Human Life Studies, Tokushima Bunri University, Tokushima 770-8514, Japan; 5 Kato Clinic of Internal Medicine, Tokyo 125-0054, Japan; norikokato.0131@gmail.com (N.K.); katom@gol.com (M.K.); 6 Jiyugaoka Medical Clinic, Obihiro 080-0016, Japan; dryokoyama@yokoyamanaika.com; 7 Kurihara Clinic, Sapporo 004-0053, Japan; ykuri@yg7.so-net.ne.jp; 8 Department of Medicine, Shiga University of Medical Science, Shiga 520-2192, Japan; maegawa@belle.shiga-med.ac.jp

**Keywords:** obesity, type 2 diabetes, food group, vegetables, fruits, soybeans/soy products, sweets

## Abstract

Background: We investigated the association between various food groups and obesity in Japanese patients with type 2 diabetes. Methods: 2070 patients with type 2 diabetes who attended 26 diabetes clinics throughout Japan were analyzed and were divided into obese and non-obese groups. Intakes of food groups determined by a food frequency questionnaire were compared. Odds ratios for obesity for quartiles of individual food groups were calculated using a logistic regression model. Results: Non-obese patients consumed a larger variety of food groups than obese patients, with the diets of non-obese individuals closer to the traditional Japanese diet characterized by fish, seaweed, and soybeans/soy products. Among 21 food groups, low vegetable intake and high sweets intake were the most strongly associated with obesity in both men and women. Low intake of both fruits and vegetables and the combination of high intake of sweets and low intake of fruits were associated with obesity. Conclusions: Food groups and their combinations that were strongly associated with obesity in Japanese patients with type 2 diabetes were identified. Our findings also suggested an inverse association between the traditional Japanese diet and obesity.

## 1. Introduction

Obesity is among the greatest risk factors for the development of type 2 diabetes [1,2]. In addition, obesity is well known to worsen the control of glycemia and other indexes and increase the risk of complications and death associated with type 2 diabetes [3,4]. As in other regions, in East Asia, which has the largest population in the world, the prevalence of obesity is increasing due to the westernization of dietary habits [5,6]. The prevalence of type 2 diabetes is also increasing [7,8].

Achieving weight loss among obese patients with type 2 diabetes improves many surrogate markers for the development of complications such as abnormal blood glucose, serum lipid, and blood pressure values [9]. Since dietary intake is related to the development of obesity, the relationship between obesity and various food groups has been investigated in several countries [10,11,12,13,14]. For example, an inverse relationship was observed between vegetable and fruit intake and weight-related outcomes [15,16]. Moreover, a randomized trial revealed that both a low-calorie diet and a low-carbohydrate diet resulted in a greater weight loss than a conventional diet [17,18,19,20]. However, adherence to the regimens described was poor, and attrition was high. Although the Mediterranean diet, which includes an abundance of whole grains, fruits, vegetables, seafood, beans, and nuts, could lower body weight [21,22], the value of its application in various races is not certain. In fact, the American Diabetes Association (ADA) reported that there is not a “one-size-fits-all” eating pattern for individuals with diabetes and recommends that meal planning should be individualized [23,24].

The Look AHEAD (Action for Health in Diabetes) study showed that an intensive lifestyle intervention, including diet, did not reduce the incidence of cardiovascular events in overweight or obese patients with type 2 diabetes [25]. However, in another study of an intensive lifestyle intervention, participants who lost ≥10% of their body weight had reduced mortality relative to diabetes support and education [26]. However, there have been few reports that we are aware of on the association between the intake of various food groups and their combinations and obesity in a large number of patients with type 2 diabetes. Additionally, few reports have precisely quantified food intake, which is essential in clinical settings.

Therefore, we analyzed the association between all food groups that could be identified and obesity in a large number of Japanese patients with type 2 diabetes.

## 2. Materials and Methods

This cross-sectional study was conducted on outpatients with type 2 diabetes treated at 26 clinics that participated in the Japan Diabetes Clinical Data Management Study Group (JDDM). The details of JDDM and CoDic software (Novo Nordisk Pharm Ltd., Tokyo, Japan) were described in a previous publication [27]. Briefly, CoDic software was used to identify the clinical characteristics of the study participants and their prescribed antihyperglycemic agents obtained from the JDDM database for the period from December 2014 to December 2019.

During the study period, the participating clinics distributed lifestyle questionnaires, and the data obtained were analyzed for 2070 patients with type 2 diabetes. Food intake was assessed by the Food Frequency Questionnaire based on food groups (FFQg) [28]. We used a standardized software program designed for population-based surveys and nutrition counseling in Japan (Eiyo-kun; Kenpakusha Co., Ltd., Tokyo, Japan) to calculate nutrient and food intakes [29]. Physical activity was calculated using the Japanese version of the International Physical Activity Questionnaire (IPAQ) short form [30,31].

The categorical variables were expressed as numerals and percentages and were compared with chi-square (χ2) tests. Continuous variables were expressed as the mean ± standard deviation (SD) or the median and interquartile range. Based on distribution, the continuous variables were compared using the unpaired Student’s t-test or Mann–Whitney U test for two-group comparisons and χ2 tests for four-group comparisons. Multiple logistic regression analysis identified variables related to obesity, which was defined as a body mass index (BMI) ≥ 25 kg/m^2^. Intake of specific food groups was expressed in quartiles.

Statistical analyses were performed using SPSS software version 27.0 (IBM Corp., Armonk, NY, USA). Differences were considered to be statistically significant at *p* < 0.05.

All of the procedures that involved human participants were performed in accordance with the ethical standards of the JDDM, Niigata University and Health Research Involving Human Subjects in Japan, and the 1964 Helsinki Declaration and its later amendments or comparable ethical standards. Written informed consent was obtained from all of the study participants.

## 3. Results

The characteristics of the man and women participants in both the non-obese group (BMI < 25) and the obese group (BMI ≥ 25) are shown in Table 1. The obese group was significantly younger than the non-obese group by approximately 7 years. In men, the HbA1c level in the obese group was significantly higher (0.4%) than in the non-obese group, but the difference was not significant in women.

Although the differences in BMI between the obese and non-obese participants were approximately 6 kg/m^2^ for men and 8 kg/m^2^ for women, energy intake and the carbohydrate-to-energy ratio did not differ significantly between man and women obese and non-obese participants. The fat-to-energy ratio differed significantly, although slightly (<1%), between the two groups, but only in men. Similarly, there was a significant difference in the protein-to-energy ratio between men and women, with a 0.5% difference for men between the obese and non-obese groups and an 0.8% difference for women, again <1%. On the other hand, the median amount of physical activity in the obese group compared to the non-obese group was 33% lower in men and 19% lower in women, both results with statistical significance. Stratified analysis by age and sex showed almost similar results. (Supplementary Table S1).

The intakes of food groups by the non-obese and obese participants according to sex are shown in Table 2. The intakes among both men and women of meat/processed meat, sweets, and sugar-sweetened beverages were significantly higher in the obese group than in the non-obese group. Conversely, food groups with a significantly lower intake by the obese group included not only low-energy foods such as vegetables and seaweed but also foods such as potatoes, fruits, sugar, fish/seafood, soybeans/soy products, and milk/dairy products. In the analysis stratified by age and sex, obesity was consistently correlated with intake of green and yellow vegetables and sweet foods (Supplementary Table S2).

The amounts of 21 food groups were divided into quartiles and adjusted for age, energy intake, current smoking, current alcohol use, physical activity, diabetes duration, use of oral hypoglycemic agents as well as insulin use, and sex. The odds ratios (ORs) for obesity were calculated using the lowest quartile as the reference (Table 3 and Table 4).

Food groups with significant negative associations for obesity in both men and women were green and yellow vegetables (OR 0.46 for men and OR 0.49 for women) and other vegetables (OR 0.53 for men and OR 0.39 for women). The ORs for total vegetables, that is, green-yellow vegetables and other vegetables combined, were 0.44 for men and 0.38 for women, both of which were significant negative associations. On the other hand, sweets were significantly and positively associated with ORs of 2.58 for men and 2.11 for women. Food groups with significant differences between quartiles only in men were fruits (OR 0.52), soybeans/soy products (OR 0.65), and sugar-sweetened beverages (OR 2.35), while there were significant differences in quartiles for fish/seafood (OR 0.51) and sugar (OR 0.54) only in women.

**Figure 1:**
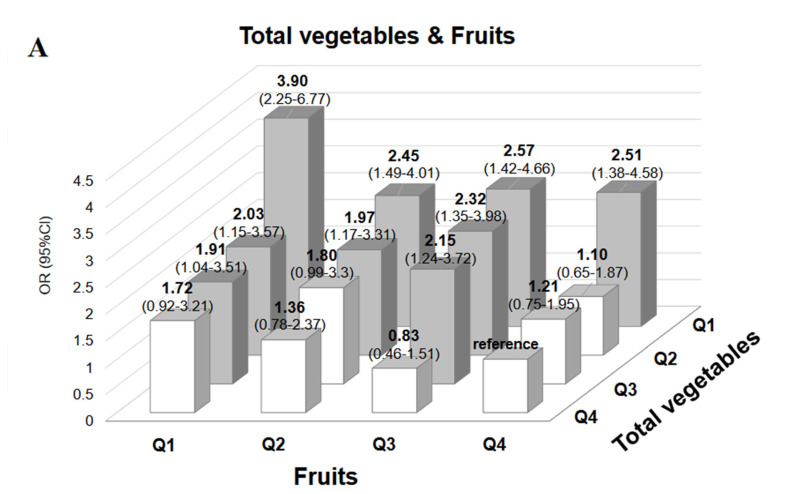

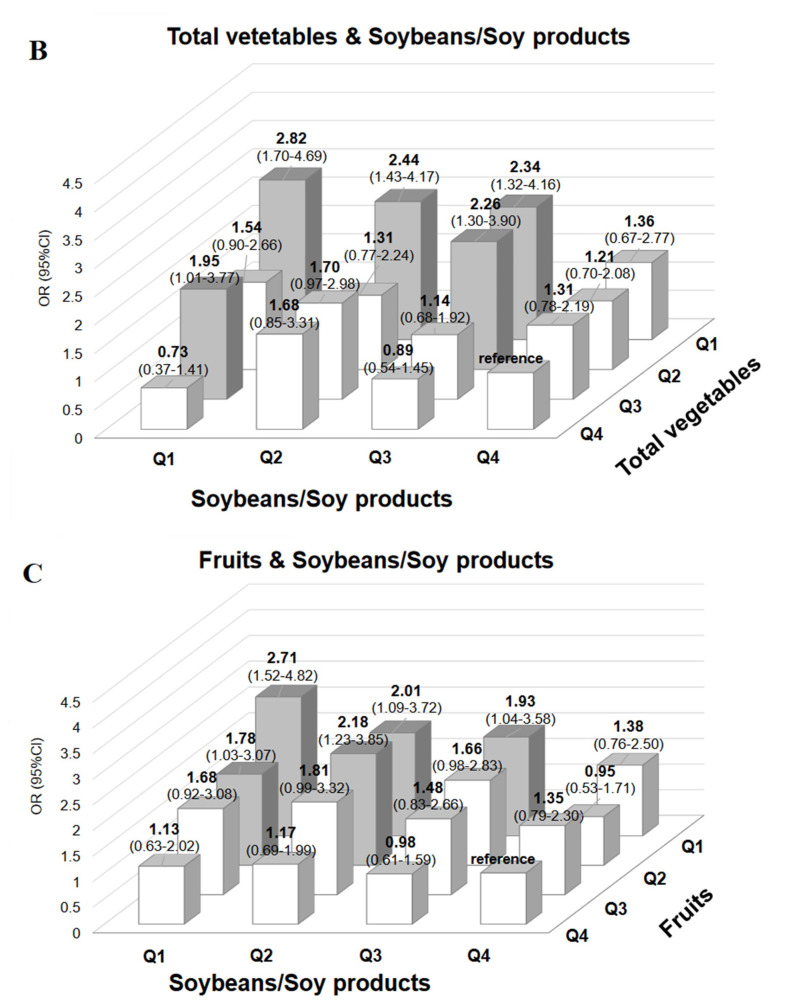

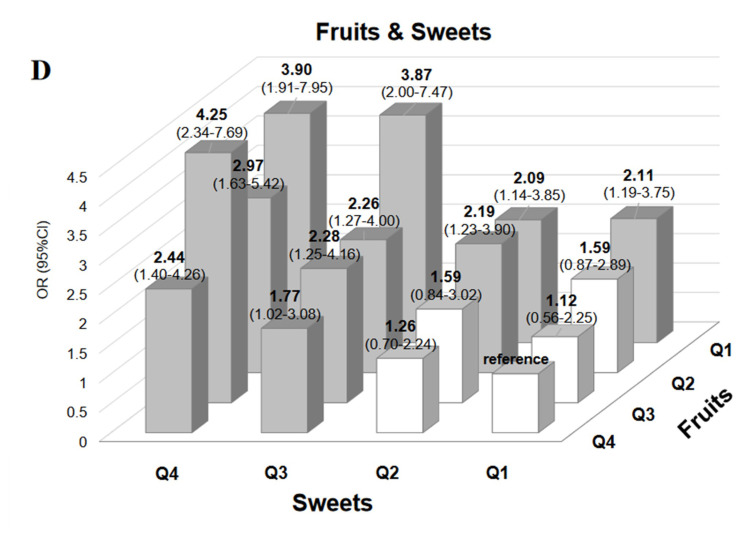
Odds ratios for obesity for four combinations of quartiles of each of food groups. (**A**). Odds ratios for obesity according to the combination of vegetables and fruits in quartiles by multi-logistic regression analysis. To investigate the interaction between vegetables and fruits in relation to obesity, 16 groups were formed by combining vegetables and fruits by quartiles. Multiple logistic regression analysis was used to investigate the odds ratios (OR) for obesity, with the reference group being the top quartile. (**B**). Odds ratios for obesity according to the combination of vegetables and soybeans/soy products in quartiles by multi-logistic regression analysis. To investigate the interaction between vegetables and soybeans/soy products in relation to obesity, 16 groups were formed by combining vegetables and soybeans/soy products by quartiles. Multiple logistic regression analysis was used to investigate the odds ratios (OR) for obesity, with the reference group being the top quartile. (**C**). Odds ratios for obesity according to the combination of fruits and soybeans/soy products in quartiles by multi-logistic regression analysis. To investigate the interaction between fruits and soybeans/soy products in relation to obesity, 16 groups were formed by combining fruits and soybeans/soy products by quartile. Multiple logistic regression analysis was used to investigate the odds ratios (OR) for obesity, with the reference group being the top quartile. (**D**). Odds ratios for obesity according to the combination of fruits and sweets in quartiles by multi-logistic regression analysis. To investigate the interaction between fruits and sweets in relation to obesity, 16 groups were formed by combining fruits and sweets by quartiles. Multiple logistic regression analysis was used to investigate the odds ratios (OR) for obesity, with the reference group for fruits being the top quantile and that for sweets being the bottom quantile.

We investigated if there were additive or synergistic associations among four food groups that were strongly associated with obesity, i.e., total vegetables, fruits, soybeans/soy products, and sweets. Since there was no difference in the strength of association with obesity between green-yellow vegetables and other vegetables, we combined the two as “total vegetables”. Figure 1 shows ORs for obesity for four combinations of quartiles of each of these food groups.

Total vegetables and fruits comprised the combination with the highest association with obesity; the OR for obesity for the lowest quartiles of these food groups was 3.9. However, the ORs for obesity were significantly increased even for the combination of total vegetable intake and fruit intake in Q3. For the combination of the lowest quartiles of total vegetables and soybeans/soy products, the OR for obesity was high at 2.82. The OR for obesity for the combination of the lowest quartiles of soybeans/soy products and fruits was 2.71. On the other hand, for sweets and fruits, the OR for obesity according to the highest intake of sweets and the lowest intake of fruits was 3.90 compared with the lowest intake of sweets and the highest intake of fruits.

## 4. Discussion 

The development of obesity is not only related to energy intake but also to specific components of foods and nutrients [32], which was the rationale for this study to determine which food groups were associated with obesity. Our results showed that the energy intake in the non-obese group was comparable to that in the obese group. The obese group consumed more high-energy foods than the non-obese groups, although in small volumes (i.e., high energy density [33]), such as meat/processed meat, sweets, and sugar-sweetened beverages. Conversely, the non-obese group consumed more low-energy-dense foods such as vegetables, fruits, seaweed, fish/seafood, and soybeans/soy products.

These results are consistent with reports that show that the decreased consumption of legumes, coarse grains, and vegetables and the increased consumption of processed foods such as oils, sugar-sweetened beverages, and animal products were important factors in the global epidemic of overweight and obesity [34]. The dietary composition in our non-obese group was close to a recommended dietary pattern for the prevention and management of type 2 diabetes, which is rich in whole grains, fruits, vegetables, nuts, and legumes but low in refined grains, red/processed meats, and juices [35].

In our Japanese patients with type 2 diabetes, it was shown that compared to obese patients, non-obese patients ingested a wider variety of food groups and tended to have a dietary pattern close to the traditional “Japanese diet”, which is characterized by large amounts of fish, seaweed, and soybeans. It was reported that the Japanese diet is related to a low mortality rate for cardiovascular diseases [36,37].

A further feature of foods frequently consumed by the non-obese group was that the preparation and cooking required much more effort, such as for vegetables, fruits, seaweed, potatoes, and fish/seafood. These foods are thought to be consumed less frequently when eating out or having take-away meals but more frequently at home. Their usage at home makes it easier to follow a diabetes diet. In our previous prospective study, we reported on food group intake and weight gain in Japanese men who underwent health checkups [38]. The higher the sugar intake, the higher the intake of vegetables, fruits, and potatoes. The reason is that in Japan, in addition to salads, vegetables are often boiled and seasoned with sugar. As a result, sugar intake tends to increase with the consumption of vegetables. The same trend was observed in this survey.

Among all of the 21 food groups examined, low vegetable intake and high sweets intake were most strongly associated with obesity in both men and women. A significant negative correlation between green-yellow vegetables and other vegetables and obesity was seen in both men and women. The threshold for a significant increase in the risk of obesity was ≥100 g for men and ≥86 g for women for green-yellow vegetables and ≥200 g for men, and ≥110 g for women for other vegetables. For fruits, there was a significant decrease in the OR for obesity in men with an intake of ≥64 g. The negative association with obesity in women was not as strong as in men. According to the Japanese National Health and Nutrition Examination Survey, 2019, the mean intakes of green-yellow vegetables in Japanese men and women were 79.8 g and 83.6 g, respectively, and those of other vegetables were 174.4 g for men and 161.3 g for women. Mean intakes of fruits were 87.6 g for men and 104.7 g for women. Thus, it would not seem very difficult for patients with diabetes to consume fruits and vegetables to the extent that exceeds the above thresholds.

A high intake of sweets was significantly associated with obesity in both men and women; however, there was a sex difference in the intake threshold, which was ≥38 g for men and ≥73 g for women. In terms of sugar-sweetened beverages, a significant association for obesity was seen only in men consuming even small amounts, such as ≥5 g. No association was observed in women. One reason could be the largely skewed proportion of individuals who refrained from eating these foods, which was in the bottom quartile, and reached as high as 74.8% for women (60.5% for men) and was therefore difficult to achieve statistical significance.

Since we found that among the 21 food groups examined, four food groups, i.e., total vegetables, fruits, soybeans/soy products, and sweets, were strongly associated with obesity, we investigated the risk of obesity related to several combinations of these four food groups to determine the potential additive associations. Combinations of low intake of total vegetables and low intake of fruits, as well as combinations of high intake of sweets and low intake of fruits, additively increased the obesity risk. The risk for obesity increased significantly in both men and women when intakes of both vegetables and fruits were in the third quartile compared with intakes in the top quartiles (Figure 1A). This suggests that both vegetables and fruits need to be consumed abundantly to avoid obesity. On the other hand, the risk for obesity was significantly elevated regardless of fruit intake by participants in the bottom quartile of intake of all vegetables, indicating that vegetable intake was more strongly associated with obesity than fruit intake.

It was reported that the higher the intake of fruits and vegetables, the lower the risk of developing diabetes [39]. A negative correlation between the amounts of fruits and vegetables consumed and BMI in people without diabetes has also been reported [40,41,42]. Although fruits are often believed to increase the risk of obesity because of their fructose content, it was shown that fruit intake is unlikely to contribute to obesity and, rather, moderately suppresses the risk [43]. However, few Japanese are aware of the daily recommended amounts of fruit and vegetable [44], and their fruit intake tends to be low compared to other countries [45]. In a study of Japanese men with type 2 diabetes over 65 years of age, a high intake of green-yellow vegetables was shown to be significantly associated with a low BMI [46]. Our results showed a significant association with a low BMI in men and women widely ranging in age that was not limited to green-yellow vegetables but to total vegetables.

The relationship between the consumption of legumes and obesity has been shown. The risk of overweight and obesity was reported to decrease by 12% for every 50 g/day increase in intake of legumes [14]. Legumes, mainly soybeans among Japanese people, have a high protein content, which is known to increase energy expenditure and may promote weight loss by inducing satiety. Soybeans are high in dietary fiber and promote a feeling of fullness [47,48,49]. Although the intake of legumes in some Western countries is reportedly low [48], their consumption in Japan is quite high as they are a staple of the traditional diet.

It is generally believed that a higher intake of sweets is associated with a greater risk of obesity. However, despite the many cross-sectional, longitudinal, and interventional studies to investigate the relationship between the consumption of sweets as snacks and obesity or weight gain, no consistent results have been obtained [50]. One reason is that the content, frequency, and the time of the intake of snacks are ambiguous in these studies. When snacking is limited to high-fat, high-sugar, energy-dense foods such as sweets and sugar-sweetened beverages, a significant positive correlation with obesity has been reported [51,52].

In the combined analysis of sweets and fruits shown in Figure 1D, most odds ratios for obesity were significantly high when sweets intake was above the median and fruit intake was below the median. In other words, if the intake of sweets did not exceed the median, the obesity risk was insignificant if fruit intake was approximately at the median. However, when fruit intake was in the bottom quartile, the risk for obesity was significantly high regardless of the amount of sweets intake. These results suggested that a moderate intake of sweets would be allowed if accompanied by the ingestion of median amounts of fruits such as half of an apple or one banana. However, even if fruit intake is increased, the association with obesity increases when sweets intake exceeds the median. Taken together, these findings suggest that reducing the intake of sweets can help with weight loss.

Our findings indicated that the amount of physical activity had a strong association with obesity since there was no difference in energy intake between the man and women obese and non-obese study participants, even though physical activity was much lower in obese than non-obese participants. Similar results were reported from Taiwan [53]. In addition, a cohort study from China involving 2488 people reported that work-related physical activity but not energy intake at baseline was an important determinant of weight gain [54]. Since obesity is known to be caused by an imbalance between energy intake and energy expenditure, the reasons for and background of the results, including whether it is a specific phenomenon seen in East Asia, are still a matter of debate.

The strength of this study is that it is a large-scale study of the association between a comprehensive list of food groups and obesity in patients with diabetes mellitus. Moreover, the additive associations with obesity in some food groups could be clarified. One limitation is its cross-sectional design, which cannot show a causal relationship. Another limitation is that the results may not be applicable to racial or ethnic groups that have different eating habits and genetic backgrounds.

## 5. Conclusions

Food groups, and their combinations, which are significantly associated with obesity for Japanese patients with type 2 diabetes, were identified. Our findings also suggest an inverse association between the traditional Japanese diet and obesity.

## Figures and Tables

**Table 1:** Characteristics of participants.

	Men (*n* = 1280)			Women (*n* = 790)		
	BMI < 25.0 (*n* = 625)	BMI ≥ 25.0 (*n* = 655)	*p*	BMI < 25.0 (*n* = 400)	BMI ≥ 25.0 (*n* = 390)	*p*
Age (years)	64.6 ± 10.5	57.3 ± 11.8	<0.001	67.7 ± 10.8	60.6 ± 13.7	<0.001
BMI (kg/m^2^)	22.4 ± 1.8	28.8 ± 3.6	<0.001	21.7 ± 2.2	29.6 ± 4.2	<0.001
Duration of diabetes (years)	12.8 ± 8.2	10.6 ± 7.1	<0.001	12.4 ± 8.3	11.1 ± 7.4	0.057
Systolic blood pressure (mmHg)	127 ± 15	129 ± 15	0.043	126 ± 18	126 ± 14	0.846
LDL cholesterol (mg/dL)	108 ± 25	106 ± 28	0.154	117 ± 36	107 ± 28	0.102
HDL cholesterol (mg/dL)	57 ± 17	50 ± 13	<0.001	64 ± 16	57 ± 14	<0.001
Triglycerides (mg/dL)	157 ± 131	192 ± 158	<0.001	133 ± 82	166 ± 103	<0.001
HbA1c (%)	7.0 ± 1.0	7.4 ± 1.2	<0.001	7.2 ± 1.1	7.3 ± 1.1	0.196
Current smoking (%)	23.4	26.4	0.009	5.5	9.7	0.072
Drinking alcohol (%)	63.5	56.9	0.016	25.8	24.9	0.777
Energy intake (kcal)	1817 ± 425	1863 ± 475	0.238	1682 ± 334	1685 ± 402	0.940
Protein (% energy)	14.9 ± 2.2	14.4 ± 2.3	0.001	15.7 ± 2.1	14.9 ± 2.1	<0.001
Fat (% energy)	28.8 ± 5.3	29.7 ± 5.7	0.001	30 ± 4.9	30.5 ± 5	0.105
Carbohydrate (% energy)	56.3 ± 6.5	55.8 ± 7.1	0.050	54.3 ± 5.7	54.6 ± 5.9	0.720
Treated by OHA and/or GLP-1RA (%)	77.0	79.5	0.314	74.4	81.1	0.044
Treated by insulin (%)	24.5	27.2	0.324	29.6	30.8	0.755
Physical activity (METs h/w) †	19.8 (8.5–44.2)	13.2 (4.4–33.3)	<0.001	14.3 (6.6–27.1)	11.6 (4.0–26.5)	0.025

Data are mean ± standard deviation or *n* (%). † Physical activity (metabolic equivalents [METs] h/w) are median. Differences in the continuous and categorical variables were analyzed by Student’s t-test or Mann–Whitney U tests and chi-square (χ2) tests, respectively. BMI, body mass index; LDL-cholesterol, low-density lipoprotein cholesterol; HDL-cholesterol, high-density lipoprotein cholesterol; OHA, oral hypoglycemic agent; GLP-1RA, GLP-1 receptor agonist.

**Table 2:** Food groups stratified by sex and obesity status.

	Men (*n* = 1280)			Women (*n* = 790)		
	BMI < 25.0 (*n* = 625)	BMI ≥ 25.0 (*n* = 655)	*p*	BMI < 25.0 (*n* = 400)	BMI ≥ 25.0 (*n* = 390)	*p*
Grains (g)	371 ± 120	380 ± 124	0.225	325 ± 84	332 ± 90	0.093
Rice (g)	263 ± 125	277 ± 128	0.024	233 ± 97	238 ± 105	0.367
Bread (g)	39 ± 36	34 ± 37	0.003	40 ± 31	36 ± 30	0.036
Noodles (g)	69 ± 58	70 ± 59	0.762	52 ± 48	58 ± 51	0.130
Potato (g)	26 ± 26	23 ± 25	0.036	36 ± 30	32 ± 28	0.039
Total vegetables (g)	236 ± 115	204 ± 109	<0.001	276 ± 109	239 ± 116	<0.001
Green-yellow vegetables (g)	82 ± 45	68 ± 43	<0.001	98 ± 45	83 ± 46	<0.001
Other vegetables (g)	155 ± 79	137 ± 77	<0.001	178 ± 73	156 ± 79	<0.001
Fruits (g)	85 ± 68	69 ± 73	<0.001	106 ± 69	88 ± 71	<0.001
Seaweed (g)	4.8 ± 3.5	4.2 ± 3.4	0.002	5.4 ± 3.7	5.0 ± 4.5	0.006
Fish/Seafood (g)	78 ± 46	71 ± 47	0.003	78 ± 41	65 ± 38	<0.001
Meat/Processed Meat (g)	76 ± 48	90 ± 54	<0.001	67 ± 40	75 ± 48	0.046
Eggs (g)	28 ± 19	29 ± 21	0.411	25 ± 16	26 ± 17	0.589
Soybeans/Soy products (g)	63 ± 43	56 ± 42	0.001	71 ± 42	63 ± 42	0.004
Milk/Dairy product (g)	138 ± 108	125 ± 112	0.004	151 ± 95	130 ± 98	<0.001
Milk (g)	86 ± 94	78 ± 95	0.112	89 ± 78	78 ± 85	0.013
Other dairy products (g)	52 ± 42	47 ± 42	0.007	62 ± 45	51 ± 40	0.001
Sugar (g)	7.1 ± 5.5	6.4 ± 5.5	0.003	9.1 ± 5.5	7.7 ± 5.3	<0.001
Nuts and seeds (g)	3.6 ± 5.8	3.2 ± 6.4	0.124	4.2 ± 6	2.8 ± 5.2	0.002
Fats and oils (g)	12.3 ± 7.7	12.6 ± 7.6	0.437	11.2 ± 7.6	11.9 ± 7.4	0.137
Seasonings and spices (g)	23 ± 12.4	24.2 ± 12.8	0.088	19.7 ± 10	19.9 ± 11	0.967
Sweets (g)	42 ± 41	53 ± 42	<0.001	45 ± 36	58 ± 44	<0.001
Sugar-sweetened beverages (g)	43 ± 107	63 ± 117	<0.001	15 ± 41	27 ± 75	<0.001
Alcoholic beverages (g)	133.9 ± 151.5	119.2 ± 160.9	0.013	23.3 ± 59.2	24.3 ± 63.8	0.822

Differences in the continuous and categorical variables were analyzed by Mann-Whitney U tests.

**Table 3:** Association between obesity and food groups according to quartiles with ranges by multivariate quartile analysis (men).

		Quartile 1 (Low)	Quartile 2	Quartile 3	Quartile 4 (High)	*p* for Trend
Grains	Range (g)	<317	<384	<428	≥428	
		reference	0.82 (0.56–1.21)	0.87 (0.59–1.28)	0.79 (0.51–1.24)	0.387
Rice	Range (g)	<193	<270	<347	≥347	
		reference	1.03 (0.70–1.52)	1.10 (0.75–1.61)	0.93 (0.63–1.38)	0.805
Bread	Range (g)	<4	<26	<60	≥60	
		reference	1.17 (0.79–1.73)	1.02 (0.70–1.49)	0.73 (0.51–1.05)	0.070
Noodles	Range (g)	<26	<51	<103	≥103	
		reference	1.94 (1.24–3.04)	1.54 (1.03–2.29)	1.73 (1.14–2.61)	0.058
Potato	Range (g)	<7	<14	<36	≥36	
		reference	1.33 (0.82–2.18)	0.81 (0.56–1.16)	0.87 (0.57–1.32)	0.197
Total vegetables	Range (g)	<136	<205	<296	≥296	
		reference	0.74 (0.51–1.07)	0.65 (0.44–0.96)	0.44 (0.29–0.65)	<0/001
Green-yellow vegetables	Range (g)	<43	<71	<100	≥100	
		reference	0.75 (0.51–1.09)	0.66 (0.44–1.01)	0.46 (0.31–0.68)	<0/001
Other vegetables	Range (g)	<86	<134	<200	≥200	
		reference	0.92 (0.64–1.34)	0.69 (0.47–1.01)	0.53 (0.36–0.78)	0.001
Fruits	Range (g)	<21	<64	<150	≥150	
		reference	0.74 (0.50–1.10)	0.60 (0.40–0.90)	0.52 (0.34–0.79)	0.001
Seaweed	Range (g)	<1.4	<4.3	<6.4	≥6.4	
		reference	1.07 (0.69–1.66)	0.89 (0.56–1.43)	0.80 (0.49–1.30)	0.145
Fish/Seafood	Range (g)	<43	<69	<97	≥97	
		reference	0.80 (0.55–1.18)	0.68 (0.47–1.00)	0.73 (0.49–1.10)	0.093
Meat/Processed Meat	Range (g)	<46	<74	<114	≥114	
		reference	0.91 (0.62–1.32)	1.02 (0.68–1.52)	1.02 (0.66–1.59)	0.789
Eggs	Range (g)	<14	<21	<43	≥43	
		reference	0.88 (0.55–1.41)	1.03 (0.71–1.52)	0.82 (0.54–1.24)	0.503
Soybeans/Soy products	Range (g)	<30	<50	<80	≥80	
		reference	0.92 (0.62–1.36)	0.76 (0.52–1.11)	0.65 (0.44–0.96)	0.020
Milk/Dairy product	Range (g)	<49	<109	<197	≥197	
		reference	1.33 (0.92–1.93)	1.14 (0.78–1.68)	0.82 (0.55–1.22)	0.263
Milk	Range (g)	<3	<73	<170	≥170	
		reference	1.58 (1.07–2.32)	1.59 (1.09–2.32)	0.95 (0.66–1.36)	0.987
Other dairy product	Range (g)	<18	<43	<70	≥70	
		reference	1.00 (0.69–1.46)	0.77 (0.52–1.12)	0.70 (0.47–1.06)	0.042
Sugar	Range (g)	<2.8	<5.4	<9.5	≥9.5	
		reference	0.81 (0.55–1.18)	0.80 (0.54–1.19)	0.98 (0.64–1.49)	0.894
Nuts and seeds	Range (g)	<0.1	<1.1	<3.6	≥3.6	
		reference	0.65 (0.44–0.96)	0.92 (0.62–1.39)	0.78 (0.50–1.20)	0.787
Fats and oils	Range (g)	<6.9	<11.3	<16.9	≥16.9	
		reference	0.97 (0.66–1.41)	1.05 (0.71–1.54)	0.69 (0.46–1.04)	0.128
Seasoning and spices	Range (g)	<15.8	<22.1	<29.6	≥29.6	
		reference	0.88 (0.60–1.30)	1.37 (0.93–2.01)	1.40 (0.92–2.11)	0.032
Sweets	Range (g)	<17	<38	<67	≥67	
		reference	1.29 (0.88–1.90)	1.84 (1.25–2.71)	2.58 (1.69–3.94)	<0.001
Sugar-sweetened beverages	Range (g)	<5	<50	<143	≥143	
		reference	1.53 (1.03–2.27)	1.96 (1.31–2.93)	2.35 (1.50–3.68)	<0.001
Alcoholic beverages	Range (g)	<10.7	<128.6	<300.0	≥300.0	
		reference	0.88 (0.61–1.26)	0.66 (0.45–0.96)	0.85 (0.59–1.20)	0.876

Logistic regression models adjusted for age, current smoking, current alcohol drinking, diabetes duration, physical activity, energy intake, treated by insulin, treated by OHA.

**Table 4:** Association between obesity and food groups according to quartiles with ranges by multivariate quartile analysis (women).

		Quartile 1 (Low)	Quartile 2	Quartile 3	Quartile 4 (High)	*p* for Trend
Grains	Range (g)	<279	<336	<390	≥390	
		reference	0.70 (0.43–1.16)	1.25 (0.75–2.08)	0.92 (0.55–1.55)	0.679
Rice	Range (g)	<174	<231	<309	≥309	
		reference	0.61 (0.36–1.03)	0.82 (0.51–1.32)	0.93 (0.56–1.54)	0.947
Bread	Range (g)	<9	<34	<60	≥60	
		reference	0.53 (0.31–0.90)	0.63 (0.36–1.11)	0.53 (0.31–0.90)	0.088
Noodles	Range (g)	<26	<51	<77	≥77	
		reference	1.22 (0.72–2.05)	1.10 (0.65–1.86)	1.38 (0.87–2.19)	0.221
Potato	Range (g)	<14	<29	<43	≥43	
		reference	0.90 (0.54–1.52)	0.91 (0.52–1.58)	0.92 (0.55–1.53)	0.797
Total vegetables	Range (g)	<171	<252	<340	≥340	
		reference	0.50 (0.31–0.83)	0.51 (0.31–0.85)	0.38 (0.23–0.64)	0.001
Green-yellow vegetables	Range (g)	<57	<86	<125	≥125	
		reference	0.96 (0.59–1.57)	0.56 (0.34–0.93)	0.49 (0.29–0.80)	0.001
Other vegetables	Range (g)	<110	<163	<223	≥223	
		reference	0.53 (0.32–0.87)	0.49 (0.29–0.81)	0.39 (0.24–0.65)	<0/001
Fruits	Range (g)	<32	<86	<150	≥150	
		reference	0.99 (0.60–1.63)	1.74 (0.93–3.25)	0.63 (0.38–1.05)	0.078
Seaweed	Range (g)	<2.1	<4.3	<7.1	≥7.1	
		reference	0.70 (0.42–1.18)	1.00 (0.59–1.68)	0.68 (0.41–1.14)	0.325
Fish/Seafood	Range (g)	<44	<66	<95	≥95	
		reference	0.68 (0.42–1.11)	0.78 (0.47–1.29)	0.51 (0.30–0.88)	0.036
Meat/Processed Meat	Range (g)	<40	<63	<91	≥91	
		reference	1.18 (0.73–1.91)	0.80 (0.48–1.33)	1.24 (0.70–2.20)	0.869
Eggs	Range (g)	<14	<21	<36	≥36	
		reference	1.57 (0.90–2.74)	1.24 (0.75–2.07)	1.33 (0.76–2.33)	0.564
Soybeans/Soy products	Range (g)	<35	<60	<90	≥90	
		reference	1.14 (0.69–1.87)	0.90 (0.55–1.46)	0.76 (0.44–1.29)	0.215
Milk/Dairy product	Range (g)	<63	<122	<205	≥205	
		reference	1.14 (0.71–1.85)	1.00 (0.60–1.66)	0.63 (0.37–1.07)	0.074
Milk	Range (g)	<12	<73	<170	≥170	
		reference	1.65 (1.00–2.74)	1.42 (0.88–2.29)	0.80 (0.49–1.30)	0.372
Other dairy product	Range (g)	<25	<53	<79	≥79	
		reference	1.55 (0.96–2.48)	1.11 (0.67–1.84)	0.90 (0.54–1.48)	0.396
Sugar	Range (g)	<4.4	<7.3	<11.3	≥11.3	
		reference	0.73 (0.44–1.21)	0.57 (0.35–0.95)	0.54 (0.31–0.94)	0.018
Nuts and seeds	Range (g)	<0.3	<1.4	<3.9	≥3.9	
		reference	1.05 (0.65–1.71)	0.89 (0.54–1.47)	0.70 (0.40–1.21)	0.150
Fats and oils	Range (g)	<5.9	<10.3	<15.6	≥15.6	
		reference	1.21 (0.75–1.97)	2.10 (1.27–3.49)	1.37 (0.81–2.34)	0.076
Seasoning and spices	Range (g)	<12.8	<18.8	<25.1	≥25.1	
		reference	0.73 (0.44–1.19)	0.83 (0.50–1.37)	1.18 (0.69–2.04)	0.446
Sweets	Range (g)	<21	<42	<73	≥73	
		reference	1.10 (0.67–1.80)	1.38 (0.84–2.28)	2.11 (1.22–3.67)	0.007
Sugar-sweetened beverages	Range (g)	<7	<36	<79	≥79	
		reference	2.14 (1.11–4.14)	1.46 (0.80–2.69)	1.07 (0.57–2.01)	0.285
Alcoholic beverages	Range (g)	<8.6	<42.9	<85.7	≥85.7	
		reference	0.57 (0.29–1.15)	0.72 (0.38–1.34)	0.88 (0.48–1.60)	0.334

Logistic regression models adjusted for age, current smoking, current alcohol drinking, diabetes duration, physical activity, energy intake, treated by insulin, treated by OHA.

## Data Availability

We are unable to provide an anonymized dataset containing the underlying data used to create the figures and tables because these data are the private property of the JDDM Study Group. Making it available to anyone in the general public would cause a loss of ownership of the data by the JDDM Study Group.

## Supplementary Materials

The following supporting information can be downloaded at: https://www.mdpi.com/article/10.3390/nu14153034/s1. Table S1. Characteristics of participants between under 65 and over 65 age by sex, Table S2 Food groups stratified and obesity status between under 65 and over 65 age by sex, Supplemental list of participants.
