# Supplementary material for: Association between Obesity and Intake of Different Food Groups among Japanese with Type 2 Diabetes Mellitus—Japan Diabetes Clinical Data Management Study (JDDM68)
Source: Nutrients. 2022 Jul 24;14(15):3034. doi: 10.3390/nu14153034 (PMC9331232; doi:10.3390/nu14153034)
Supplement: Supplementary file 1 [file nutrients-14-03034-s001.zip › nutrients-1780123-supplementary.pdf]

Supplementary materials

Supplementary Table S1. Characteristics of participants between under 65 and over 65 age by sex.

	men						women					
	<65years			≥65years			<65years			≥65years		
	BMI<25 (n=294)	BMI≥25 (n=465)	p	BMI<25 (n=331)	BMI≥25 (n=190)	p	BMI<25 (n=144)	BMI≥25 (n=224)	p	BMI<25 (n=256)	BMI≥25 (n=166)	p
Age (years)	55.7±7.0	51.5±8.2	<0.001	72.6±5.6	71.5±5.8	0.011	56.5±7.7	51.6±10.9	<0.001	74±6.1	72.6±5.6	0.044
BMI (kg/m2)	22.5±1.8	29.4±3.9	<0.001	22.3±1.8	27.5±2.2	<0.001	21.5±2.3	30.5±4.6	<0.001	21.8±2.2	28.5±3.3	<0.001
Duration of diabetes (years)	9.8±6.8	9.4±6.2	<0.001	15.4±8.5	13.6±8.1	0.022	9.0±6.4	9.4±6.6	0.596	14.3±8.7	13.4±7.8	0.284
Systolic blood pressure (mmHg)	125±15	128±15	0.035	127±15	130±14	0.155	124±18	124±13	0.993	128±17	129±15	0.578
LDL cholesterol (mg/dl)	112±28	107±29	0.052	105±23	99±25	0.298	122±38	108±28	0.053	113±34	106±27	0.281
HDL cholesterol (mg/dl)	56±16	49±12	<0.001	57±17	51±15	0.003	69±17	58±15	<0.001	63±16	56±13	0.001
Triglycerides (mg/dl)	174±148	200±179	0.015	139±109	172±89	<0.001	130±92	171±121	<0.001	135±76	160±75	0.001
HbA1c (%)	7.2±1.3	7.5±1.3	<0.001	6.9±0.7	7.1±0.8	0.006	7.4±1.3	7.4±1.3	0.928	7.1±0.9	7.2±0.9	0.164
Current smoking (%)	31.0	31.8	0.673	16.6	13.2	0.071	10.4	12.5	0.53	2.7	6.0	0.164
Drinking alcohol (%)	67.3	55.3	0.001	60.1	61.1	0.834	31.3	30.4	0.856	22.7	17.5	0.198
Energy intake (kcal)	1821±413	1854±484	0.696	1813±435	1883±452	0.083	1666±341	1714.6±428	0.226	1692±330	1644±360	0.135
Protein (% energy)	14.8±2.5	14.2±2.2	0.013	14.9±1.9	15.0±2.3	0.866	15.2±2.2	14.7±2.1	0.028	16.0±1.9	15.2±1.9	<0.001
Fat (% energy)	29.6±5.7	30.3±6	0.023	28.1±4.9	28.4±4.8	0.558	31.1±5.9	31.8±5.0	0.251	29.3±4.1	28.8±4.4	0.249
Carbohydrate (% energy)	55.7±7.1	55.5±7.4	0.256	56.9±6	56.6±6.1	0.576	53.7±7.0	53.5±6.0	0.843	54.7±4.9	56±5.5	0.013
Treated by OHA and/or GLP-1RA (%)	79.8	78.3	0.639	74.3	82.6	0.045	74.1	79.3	0.294	74.6	83.6	0.051
Treated by insulin (%)	21.0	23.5	0.474	27.7	35.9	0.076	25.9	27.8	0.720	31.9	35	0.556
Physical activity (METs・h/w)†	16.5 (7.1-40.5)	11.6 (4.0-27.4)	<0.001	23.1 (11.0-46.2)	19.0 (6.6-43.8)	0.078	17.1 (6.6-31.0)	11.9 (4.1-26.6)	0.025	13.2 (6.6-23.2)	11.4 (3.9-26.8)	0.139

Data are mean ± standard deviation or n (%). † Physical activity (metabolic equivalents [METs]・h/w) are median. Differences in the continuous and categorical variables were analyzed by Student's t test or Mann-Whitney U tests and chi-square (χ2) tests, respectively.

BMI, body mass index; LDL-cholesterol, low-density lipoprotein cholesterol; HDL-cholesterol, high-density lipoprotein cholesterol; OHA, oral hypoglycemic agent; GLP-1RA, GLP-1 receptor agonist

Supplementary Table S2. Food groups stratified and obesity status between under 65 and over 65 age by sex.

	men						women					
	<65yearas			≥65years			<65years			≥65years		
	BMI<25 (n=294)	BMI≥25 (n=465)	p	BMI<25 (n=331)	BMI≥25 (n=190)	p	BMI<25 (n=144)	BMI≥25 (n=224)	p	BMI<25 (n=256)	BMI≥25 (n=166)	p
Grains (g)	376±128	388±127	0.454	367±111	362±115	0.911	325±92	328±92	0.631	325±78	336±87	0.057
Rice (g)	273±136	288±132	0.113	254±114	248±114	0.979	235±109	232±103	0.829	231±89	245±107	0.074
Bread (g)	35±37	32±34	0.332	43±35	39±44	0.044	41±32	37±31	0.145	39±30	34±28	0.099
Noodles (g)	68±58	67±58	0.764	70±59	75±63	0.203	49±48	59±54	0.103	54±49	57±47	0.487
Potato (g)	22±25	21±21	0.530	29±27	30±31	0.696	25±22	28±26	0.783	42±32	37±29	0.187
Total vegetables (g)	222±108	196±107	<0.001	248±120	226±110	0.066	274±115	233±114	<0.001	278±106	247±118	0.002
Green-yellow vegetables (g)	75±43	64±41	<0.001	87±46	77±44	0.029	97±49	80±45	<0.001	99±43	86±48	<0.001
Other vegetables (g)	147±75	132±77	0.002	161±82	148±74	0.151	177±77	153±79	0.002	179±71	162±79	0.008
Fruits (g)	63±60	54±65	0.008	105±68	104±80	0.453	84±64	74±67	0.090	119±68	106±72	0.028
Seaweed (g)	4.5±3.3	3.8±3.2	0.004	5±3.6	5.3±3.8	0.446	4.5±3.3	4.6±3.9	0.600	6±3.9	5.6±5.2	0.032
Fish/Seafood (g)	74±47	64±44	0.003	81±46	89±51	0.131	63±36	57±37	0.074	87±41	76±37	0.007
Meat/Processed Meat (g)	86±52	97±55	0.006	68±43	73±45	0.232	79±46	86±52	0.289	60±34	60±37	0.763
Eggs (g)	30±21	29±22	0.843	27±18	28±19	0.639	23±14	28±18	0.101	25±17	23±14	0.258
Soybeans/Soy products (g)	61±44	53±41	0.007	65±42	64±43	0.539	65±42	62±43	0.662	74±41	64±40	0.006
Milk/Dairy product (g)	125±111	116±109	0.164	149±104	147±116	0.458	138±100	122±90	0.120	158±91	139±106	0.004
Milk (g)	76±100	72±95	0.988	95±87	93±95	0.610	81±79	71±78	0.288	94±77	88±93	0.115
Other dairy products (g)	48±41	44±41	0.052	54±43	54±44	0.694	57±41	51±43	0.073	64±47	51±37	0.007
Sugar (g)	5.5±4.3	5.4±4.8	0.227	8.5±6	8.8±6.4	0.711	6.7±4.2	6.5±4.6	0.445	10.4±5.7	9.3±5.7	0.018
Nuts and seeds (g)	3.6±5.6	2.8±5.1	0.005	3.6±6	4.2±8.7	0.088	4.4±6.1	2.7±5.5	0.010	4.1±6	3±4.6	0.111
Fats and oils (g)	12.7±7.9	13.2±7.7	0.351	12±7.6	11.4±7.2	0.325	12±8.5	12.7±7.7	0.181	10.7±6.9	10.8±7	0.982
Seasonings and spices (g)	22.7±12.6	23.5±12.1	0.267	23.3±12.2	26±14.1	0.049	18.2±8.8	19.5±11.3	0.394	20.6±10.5	20.5±10.5	0.914
Sweets (g)	42±44	53±42	<0.001	42±39	52±43	0.005	49±35	61±44	0.012	44±36	53±43	0.014
Sugar-sweetened beverages (g)	46±103	66±126	<0.001	40±110	53±92	<0.001	18±42	37±93	0.006	13±41	13±35	0.068
Alcoholic beverages (g)	147±157	110±158	<0.001	123±146	143±166	0.363	27±59	32±76	0.994	21±59	13±40	0.179

Differences in the continuous and categorical variables were analyzed by Mann-Whitney U tests.

Supplemental list of participants

Koichi Iwasaki, Iwasaki Medical Clinic, Yamaguchi, Japan

Kazuhiro Miyazawa, Miyazawa Clinic, Hokkaido, Japan

Osamu Tomonaga, Tomonaga Clinic, Tokyo, Japan

Katsuya Yamazaki, Kawai Clinic, Ibaraki, Japan

Noriharu Yagi, Yagi Internal Medicine Clinic, Okinawa, Japan

Masae Minami, Minami Masae Internal Medicine Clinic, Fukuoka, Japan

Hideki Wakamatsu, Wakamatsu Internal Medicine Clinic, Akita, Japan

Hidekatsu Sugimoto, Sugimoto Clinic, Kitakyushu, Japan

Tomohiro Iizumi, Iizumi Internal Medicine Clinic, Ibaraki, Japan

Masato Takaki, Takaki Internal Medicine Clinic, Niigata, Japan

Hiroshi Takeda, Takeda Clinic, Kanagawa, Japan

Akira Okada, Okada Internal Medicine Clinic, Fukuoka, Japan

Nobuichi Kuribayashi, Misaki Internal Medicine Clinic, Chiba, Japan

Michiko Tyousa, Wakamatsu Kinen Hospital, Kagoshima, Japan

Akiko Hosokawa, Hikari Clinic, Nara, Japan

Fuminobu Okuguchi, Okuguchi Clinic of internal medeicine, Miyagi, Japan

Keiko Arai, Arai Clinic, Yokohama, Japan

Mikihiko Kudo, Kudo Internal Medicine Clinic, Aomori, Japan

Masahiro Iwamoto, Iwamoto Internal Medicine Clinic, Kagawa, Japan

Hiroshi Takamura, Takamura Internal Medicine Clinic, Tokyo, Japan

Mariko Oishi, Oishi Internal Medicine Clinic, Kyoto, Japan

Hiroaki Seino, Seino Internal Medicine Clinic, Fukushima, Japan

Tetsuya Moriai, Megumino Hospital, Hokkaido, Japan
